# Increased Plin2 Expression in Human Skeletal Muscle Is Associated with Sarcopenia and Muscle Weakness

**DOI:** 10.1371/journal.pone.0073709

**Published:** 2013-08-15

**Authors:** Maria Conte, Francesco Vasuri, Giovanni Trisolino, Elena Bellavista, Aurelia Santoro, Alessio Degiovanni, Ermanno Martucci, Antonia D’Errico-Grigioni, Daniela Caporossi, Miriam Capri, Andrea B. Maier, Olivier Seynnes, Laura Barberi, Antonio Musarò, Marco V. Narici, Claudio Franceschi, Stefano Salvioli

**Affiliations:** 1 Department of Experimental, Diagnostic and Specialty Medicine and Interdepartmental Centre “L. Galvani” (CIG), University of Bologna, Bologna, Italy; 2 “F. Addarii” Institute of Oncology and Transplant Pathology, S. Orsola-Malpighi Hospital, University of Bologna, Bologna, Italy; 3 Reconstructive Hip and Knee Joint Surgery, Istituto Ortopedico Rizzoli, Bologna, Italy; 4 Department of Health Science, University of Rome “Foro Italico”, Rome, Italy; 5 Department of Gerontology and Geriatrics, Leiden University Medical Center, Leiden, The Netherlands; 6 Norwegian School of Sport Sciences, Oslo, Norway; 7 Institute Pasteur Cenci-Bolognetti, DAHFMO-unit of Histology and Medical Embryology, IIM, Sapienza University of Rome, Rome, Italy; 8 School of Graduate Entry Medicine and Health, Division of Clinical Physiology, Derby Royal Hospital, University of Nottingham, Derby, United Kingdom; Stem Cell Research Institute, Belgium

## Abstract

Human aging is associated with a progressive loss of muscle mass and strength and a concomitant fat accumulation in form of inter-muscular adipose tissue, causing skeletal muscle function decline and immobilization. Fat accumulation can also occur as intra-muscular triglycerides (IMTG) deposition in lipid droplets, which are associated with perilipin proteins, such as Perilipin2 (Plin2). It is not known whether Plin2 expression changes with age and if this has consequences on muscle mass and strength. We studied the expression of Plin2 in the vastus lateralis (VL) muscle of both healthy subjects and patients affected by lower limb mobility limitation of different age. We found that Plin2 expression increases with age, this phenomenon being particularly evident in patients. Moreover, Plin2 expression is inversely correlated with quadriceps strength and *VL* thickness. To investigate the molecular mechanisms underpinning this phenomenon, we focused on IGF-1/p53 network/signalling pathway, involved in muscle physiology. We found that Plin2 expression strongly correlates with increased p53 activation and reduced IGF-1 expression. To confirm these observations made on humans, we studied mice overexpressing muscle-specific IGF-1, which are protected from sarcopenia. These mice resulted almost negative for the expression of Plin2 and p53 at two years of age. We conclude that fat deposition within skeletal muscle in form of Plin2-coated lipid droplets increases with age and is associated with decreased muscle strength and thickness, likely through an IGF-1- and p53-dependent mechanism. The data also suggest that excessive intramuscular fat accumulation could be the initial trigger for p53 activation and consequent loss of muscle mass and strength.

## Introduction

Human aging is characterized by increased levels of physical disability due at least in part to loss of muscle strength. This loss depends on both decrease in muscle mass and accumulation of inter-muscular adipose tissue (IMAT) [1,2]. In particular, emerging evidence suggests that high level of IMAT contributes to the decline of muscle quality, predicting sarcopenia and increasing risk of mobility impairment [3,4]. Nevertheless, the precise mechanisms leading to age-related loss of muscle quality and strength are far from being elucidated. Many studies have focused on the role of IMAT, as it is known to be a source of inflammatory mediators [5], while much less is known about the possible role of intracellular lipid deposition, which occurs in form of lipid droplets (LDs). LDs are vesicles formed by a phospholipid monolayer whose dynamics appears to be determined by a family of proteins named Perilipins (Plins), previously referred to as PAT proteins, which play a critical role in regulating intracellular lipid storage and mobilization [6,7]. The PAT family consists of five specific members: Perilipin (Plin1), Adipocyte differentiation-related protein (ADRP or also referred to as Plin2), Tail-interacting protein of 47 kDa (TIP47 or Plin3), S3-12 (Plin4) and OXPAT (Plin5). The expression patterns of the five Perilipins vary in different tissues. For instance, Plin1 expression is nearly exclusive of adipocytes, whereas Plin2 has been reported to be a marker for LDs in human skeletal muscle [8,9], and its content is closely related to intramuscular triglycerides [10–12]. Plin2 is mostly expressed in the type I fibres of skeletal muscle, which contain more fat than the type II fibres and where Plin2 promotes the uptake of fatty acids and their storage as triacylglycerols [13,14].

The exact function of Plin2 remains to be clearly defined, nevertheless, recent findings suggest that Plin2 is essential for lipid storage in skeletal muscle by enhancing the partitioning of excess fatty acids toward triglycerol storage in lipid droplets, thereby blunting lipotoxicity-associated insulin resistance [13,14]. Since insulin is a potent anti-proteolytic agent, the development of insulin resistance likely contributes to the loss of muscle mass [15]. Recent data indicate that Plin2 is strictly associated to LDs [16] and thus can be an indicator of intramuscular triglyceride (IMTG) deposition; moreover, increased levels of Plin2 are related to mechanisms promoting IMTG utilization during exercise and to improvements in insulin sensitivity [17]. It is not known whether Plin2 expression is modified with age in human skeletal muscle and whether this can be associated with alterations of muscle mass and strength. Therefore the objective of this study was to investigate the different expression levels of Plin2 in skeletal muscle from subjects of different age (from 20 to over 80 years), either healthy people or patients affected by mobility-limiting pathologies (osteoarthritis) leading to sedentary life style. In the framework of the EU 7^th^ Program Project MYOAGE (“Understanding and combating human age-related muscle weakness”), we analysed Plin2 expression and distribution in Vastus lateralis (VL) muscle and associated Plin2 expression with measurements of muscle strength and thickness. To substantiate the data on humans, an animal model protected from age-related sarcopenia because of an overexpressing IGF-1 at the level of muscle was also studied [18].

## Materials and Methods

### Subjects and Ethics Statement

In the present study, within the framework of the EU Project “MYOAGE”, we recruited and analysed two groups of Caucasian subjects, either healthy subjects or patients with limited lower limb mobility.

The study protocol was approved by the local ethics committees responsible for the recruiting units: Istituto Ortopedico Rizzoli and Sant’Orsola-Malpighi University Hospital (Bologna–Italy) for patients and Leiden University Medical Center for healthy subjects (Leiden–The Netherlands) and was carried out in accordance with the current revision of the Declaration of Helsinki concerning medical research in humans, and with current Good Clinical and Laboratory Practice Guidelines of European Medicine Agency.

For the healthy group, 15 young (aged 18 to 30 years) and 30 old (aged 70 to 80 years) subjects were recruited in Leiden, The Netherlands (table S1). Application of exclusion criteria was aimed to select healthy participants, minimizing the confounding effect of disease on muscle mass, i.e. dependent living situation, unable to walk a distance of 250 m, presence of comorbidity (neurologic disorders; metabolic diseases; rheumatic diseases; recent malignancy; heart failure; severe chronic obstructive pulmonary disease (COPD)), haemocoagulative syndromes, use of specified medication (immunosuppressive drugs; insulin, anticoagulation), immobilization for one week during last three months, and orthopaedic surgery during the last two years or still causing pain or functional limitation. Both young and old subjects were involved in moderate intensity activities in the daily life, excluding those involved in competitive sports and master athletes.

For the patients group, 20 young (aged 24 to 38 years) subjects and 20 old (aged 70 to 95 years) subjects scheduled for total hip arthroplasty were recruited in Bologna, Italy (table S2). Inclusion criteria were age > 20 years and an ability to provide informed consent for the study and to cooperate with study personnel, while exclusion criteria were chronic kidney or liver diseases, bleeding disorders, severe diabetes mellitus, rheumatic diseases other than osteoarthritis, neuromuscular disorders, malignancies and systemic infections, chronic steroid use, major psychological problems or history of alcohol or drug abuse, evidence of prior surgery in the involved hip. Written informed consent was obtained from each patient before the start of the study. A thorough medical history, including systemic diseases, smoking habits and alcohol consumption, occupation and level of physical activity was taken from each patient and reported on an appropriate form. Elective unilateral total hip arthroplasty was performed by the same team at the Unit for Reconstructive Hip and Knee Surgery, Istituto Ortopedico Rizzoli.

### Quadriceps Strength Measurement

For patients recruited in Bologna isometric quadriceps strength (IQS) was measured. IQS was tested in the seated position and at the same knee angle using a Handifor^®^ dynamometer (TRACTEL S.A. Montreuil Cedex – France). After a warming up period, in which the patient received instructions about the exercise and performed a series of submaximal contractions for familiarization with the instrument, patient was asked to perform three series of 10 contractions, progressively increasing the strength developed. The highest peak torque was withheld as the MVC.

For healthy subjects (recruited in Leiden), IQS was measured with a quadriceps chair (Netherlands: Forcelink B.V., Culemborg, the Netherlands). The subjects were positioned in an upright position, with straps to fix the hips to the chair and the ankle to the force transducer at the knee angle of 90 degrees. Lever arm length was recorded. Three trials were conducted to measure maximal voluntary contraction of the quadriceps. Each trial was separated by one minute of rest. The trial with the highest force output was taken for analyses

### Muscle ultrasound measurements

Ultrasound imaging of *VL* of the affected leg were obtained using a portable ultrasound (Mylab25, Esaote, Ge nova , Italy) fitted with a 7–10 MHz linear probe. Acquisition of images was performed by an experienced and previously trained examiner. With the patient lying in supine position, the length of the thigh was measured as the distance between the greater trochanter and the distal lateral epicondyle of the femur. Cross-sectional ultrasound images were obtained between 2/3 proximal and 1/3 distal of the thigh length. Muscle thickness was measured offline, as the vertical distance between muscle superficial and deep aponeuroses at an equidistant point from right and left borders of the sagittal image.

### Biopsy sampling and analysis

For healthy subjects muscle biopsies were taken from the *VL* muscle after localised anaesthesia with 1% lidocaine, with a modified Bergstrom needle (Maastricht Instruments, Maastricht, The Netherlands) using applied suction in the morning. Muscle biopsies were taken around 10 cm of the cranial side of the patella on the lateral side of the upper leg, immediately snap-frozen in liquid nitrogen and stored at -80 °C until further analysis (real time RT-PCR and western blotting).

For patients, biopsies from the *VL* muscle and from subcutaneous adipose tissue were taken during the operation at the site of surgical incision, just distally to the proximal origin of the muscle at the level of the greater trochanter. The open surgical biopsies were immediately snap-frozen in liquid nitrogen and stored at -80 °C until further analysis (real time RT-PCR and western blotting). For the first 16 samples collected we could obtain a larger biopsy that allowed us to perform also histological and immunohistochemistry staining. To this aim the samples were treated as follows: for histochemical staining, a part of each sample was immediately embedded in OCT (Optimal Cutting Temperature) medium and then frozen for cryosectioning; for immunohistochemistry, part of each sample was fixed in formalin, embedded in paraffin (Formalin-Fixed Paraffin-Embedded (FFPE)), and routinely processed.

### Animals

FVB Wild-type (WT) and MLC/mIGF-1 mice (from Musarò Laboratories) were housed in a temperature-controlled (22° C) room with a 12: 12 hours light-dark cycle.

All mice were maintained according to the institutional guidelines of the animal facility of the unit of Histology and Medical Embryology – National Institute of Health-Italy. The experimental protocol “Characterization of factors involved in muscle diseases”, aimed at the production of transgenic animals that were used in this study, was reviewed and approved by the Institutional animal care and use committee of the Unit of Histology and Medical Embryology (University of Rome “La Sapienza”) on February 14th, 2011. The animals were sacrificed under sodium pentobarbital anaesthesia.

### RNA extraction, cDNA synthesis and quantitative real time RT-PCR

Total RNA from muscle and adipose tissue was extracted using respectively mirVANA kit (Ambion, Austin, TX, USA) and Adipose tissue RNeasy kit (QIAGEN GmbH, Germany), according to the manufactures’ instructions. After DNase treatment with TURBO DNA-free kit (Ambion, Austin, TX, USA), cDNA was synthesized using SuperScript III Reverse Transcriptase kit (Invitrogen, Carlsbad, California, USA) according to the manufactures’ instructions.

Relative quantification was performed using MESA GREEN MasterMix Plus for SYBR Assay (Eurogentec, Seraing, Belgium) by real time RT-PCR using Rotor gene Q 6000 system (QIAGEN GmbH, Germany). In brief, the following primers for the targets genes were used: Plin1 (F: 5’-ATTGCTCTGAGCTGAAGGACACCA-3’, R: 5’-AGCTCGAGTGTTGGCAGCAAATTC-3’), Plin2 (F: 5’-TGAGATGGCAGAGAACGGTGTGAA-3’, R: 5’-TTGCGGCTCTAGCTTCTGGATGAT-3’) and IGF-1 (F: 5’-GACATGCCCAAGACCCAGAAGGA-3’, R: 5’-CGGTGGCATGTCACTCTTCACTC-3’). Two different housekeeping genes, GAPDH (F: 5’-CATTGCCCTCAACGACCACTTTGT-3’, R: 5’-CATTGCCCTCAACGACCACTTTGT-3’) and 18S ribosomal RNA (F: 5’-CGTTCTTAGTTGGTGGAGCG-3’, R: 5’-CGCTGAGCCAGTCAGTGTAG-3’) were chosen for skeletal muscle and adipose tissue, respectively. Real time RT-PCR reactions were performed in duplicate in the same run and each run were repeated twice for all measurements. The mean of experiments was considered for the analysis. All reactions consisted of an initial denaturation step at 95 °C for 5 min, followed by 45 cycles of 95 °C for 15 sec and 60 °C for 60 sec. A referent sample (healthy young) was used as internal calibrator in each run. The ΔC_t_ value was calculated by subtracting the C_t_ value for housekeeping genes from the C_t_ value for the target gene of the same sample. The ΔΔC_t_ was then calculated by subtracting the ΔC_t_ value of a control subject (healthy young) from the ΔC_t_ value of the subject. Relative expression level was then determined by calculating 2-^ΔΔCt^ [19].

### Protein extraction

100 mg of frozen human muscle were lysed in TEAD buffer (Tris-HCl 20 mM pH= 7.5, EDTA 1mM, NaN_3_ 1mM, DTT 1mM) containing protease inhibitor cocktail (Sigma-Aldrich, St. Louis, MO, USA) and phosphatase Inhibitor Cocktail 2 (Sigma-Aldrich, St. Louis, MO, USA), homogenized using a motor-driven homogenizer and centrifuged at 25,000 g for 1h at 4 °C. Adipose tissue lysates were obtained by homogenizing in lysis buffer (Tris-HCl 50 mM pH= 7.6, NaCl 150mM, Triton-X 100 1%) containing protease inhibitor cocktail and phosphatase Inhibitor Cocktail 2 and centrifuging at 14,000 g for 30 min at 4 °C.

Protein extraction from quadriceps muscle of old (23-25 months) WT mice and MLC/mIGF-1 transgenic mice was performed in lysis buffer (Tris-HCl 50mM pH7.4, sodium deoxycholate 0.025%, NaCl 150mM, phenylmethylsulfonyl fluoride 1mM, Triton-X 100 1%, aprotinin 1µg/ml, leupeptin 1µg/ml, pepstatin 1µg/ml, sodium orthovanadate 1mM, sodium fluoride 1mM).

The supernatants containing the total protein extracts were quantified by Bradford’s method and tissue lysates were stored at -80 °C until analysis.

### Western blot

About 40 µg of the total protein was separated in 12% SDS-polyacrilamide gel and transferred to a nitrocellulose membrane (Trans-Blot Transfer Medium, Bio Rad, Hercules, CA), as already described [20]. After blocking in 5% non-fat dry milk/0.01% Tween 20-TBS, membranes were incubated overnight with the following antibodies: 1:1000 rabbit monoclonal D1D8 anti-perilipin1 (Cell Signalling Technology, Millipore, Beverly MA USA), 1:1000 rabbit polyclonal anti-perilipin2 (Lifespan Biosciences, Seattle WA USA), 1:1000 rabbit monoclonal C26H12 anti-PPAR-γ (Cell Signalling Technology, Millipore, Beverly MA USA), 1:500 mouse monoclonal p53 (DO-1) (Santa-Cruz Biotechnology, CA, USA), 1:500 rabbit monoclonal 7F5 anti-p53 (Cell Signalling Technology, Millipore, Beverly MA USA), 1:500 rabbit polyclonal anti-phospho-p53 (Ser20) (Cell Signalling Technology, Millipore, Beverly MA, USA). Housekeeping 1:10000 mouse monoclonal SAP.4G5 anti-βtubulin (Sigma-Aldrich, St. Louis, MO, USA) and 1:700 anti-βactin (C-11) (Santa-Cruz Biotechnology, CA, USA) were used as a loading control. The filters were incubated with each antibodies overnight at 4 °C in 5% non-fat dry milk/0.01% Tween 20 in TBS or in 5% BSA/0.01% Tween 20 in TBS for phosphorylated antibody. The secondary anti-mouse (Bio-Rad, Hercules, CA, USA), anti-rabbit horseradish peroxidase-conjugated antibody (Bio-Rad, Hercules, CA, USA) and bovine anti-goat IgG-HRP (Santa-Cruz Biotechnology, CA, USA) were used, followed by ECL detection (Santa-Cruz Biotechnology, CA, USA).

### Light microscopy and Immunohistochemistry

Sixteen muscle biopsies obtained from patients undergoing hip replacement were selected for histological and immunohistochemical (IHC) analysis, 10 males and 6 females, mean age 53.9 ± 25.8 years (range 24-94). Mean age of male patients was 50.7 ± 24.6 years, and mean age of female patients was 59.3 ± 29.3 years (p = 0.382, chi-square test).

#### Histological analysis

Histological analysis was performed in all 16 cases with Haematoxylin-Eosin stain, in order to exclude pathological conditions, such as presence of inflammatory infiltration, fibrosis and/or fibre disarray, and Oil Red O stain. Briefly, frozen sections were dehydrated in isopropyl alcohol for 1 min, and stained with a solution of 60% Oil Red O and 40% H2O for 10 min; the solution was prepared in our laboratory 1 hour before the use. Sections were then differentiated in isopropyl alcohol 60% for 4 seconds, washed in H2O, counterstained with Mayer’s haematoxylin and mounted with glycerine.

#### Immunohistochemistry

The mean major axis of the specimens used for IHC analysis was 9.51±3.38 mm (range 3.80-15.80 mm). Three µm-thick sections were cut from FFPE tissue, deparaffinized and rehydrated. Endogenous peroxydase activity was blocked by incubation in 0.3% H_2_O_2_ in methanol for 30 min. Antigen retrieval was performed by microwave heating in 10 mM citrate buffer, pH 6.0, for 20 min, according to manufacturer’s instructions. The same anti-perilipin1 and anti-perilipin2 antibodies used for WB were applied to the sections at room temperature for 1 h, followed by incubation with EnVision Plus System-HRP (Dako, Glostrup, Denmark) for 20 min and 3’, 3’ Diaminobenzidine (DAB, Dako) as chromogen for staining reaction. Slides were counterstained with Mayer’s haematoxylin. Negative controls were performed by omitting the primary antibodies.

Plin2 positivity in the muscle fibres was semi-quantitatively assessed throughout the entire surgical biopsy as follows: a 1+ staining score was attributed to those cases with scattered Plin2-positive droplets not visible at 10x magnification; a 2+ score was attributed to those cases with more intense positivity (visible at 10x magnification) due to a higher density of positive droplets, but without strongly-positive fibres; a 3+ score was attributed to those cases with at least one area of strong Plin2 positivity (intense staining at 10x magnification).

### Statistical analysis

Results are shown as mean ± s.d. unless otherwise stated. Student’s *t* test and analysis of variance (ANOVA) test were used for comparison of the means among groups (young and old healthy subjects and patients classified by age group and/or sex). The basic criteria for ANOVA, normality and homoscedasticity, have been tested by the Shapiro-Wilk, Shapiro-Francia, Skewness/Kurtosis and Bartlett’s tests. When normality or homogeneity of variance were not verified, Kruskal-Wallis or Mann-Withney tests were computed to compare groups.

Linear regression analysis has been used to identify possible correlations within a number of variables such as age, Plin2 protein expression, IHC Plin2 score, IGF-1 and p53 expression and other continuous variables (quadriceps strength/thickness ratio and quadriceps strength/BMI ratio, muscle thickness). All statistical analyses were performed using the software STATA v.9.0 (Stata Corp., Texas, USA).

## Results

**Figure 1:**
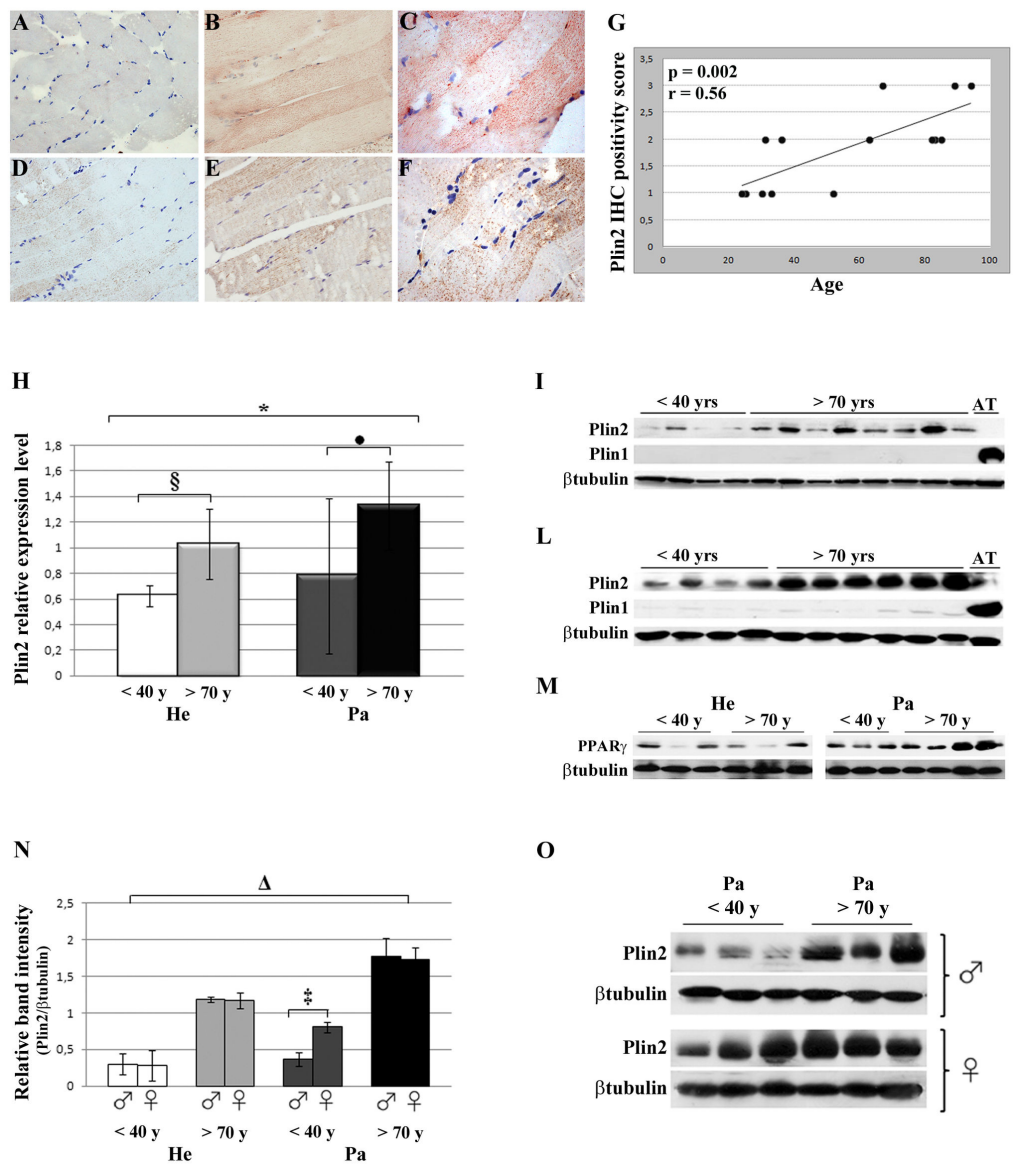
Plin2 expression changes with ageing and inactivity. (**A**–**C**) Oil Red O staining of *VL* biopsies from 3 representative patients of different age. (**D**–**F**) IHC of Plin2 in the same samples. A,D: young subject (25 yrs); B, E: late adult (63 yrs); C,F: old subject (81 yrs). Magnification 20x. (**G**) Regression graphic of Plin2 positivity *vs* age of patients. IHC positivity score was semi-quantitatively assessed. (**H**) Real time RT-PCR expression level of Plin2 in young (<40 yrs) and old (>70 yrs) healthy subjects (He) and in young (<40 yrs) and old (>70 yrs) patients (Pa). The amount of RNA in the different samples is normalized to GAPDH as reference gene and shown as relative expression (ΔΔCt). Data are expressed as mean ± st. dev. *p=0.*0003*, Kruskall–Wallis test (non-parametric ANOVA); §*p=0*.*009* and •*p=0*.*001*, Mann–Whitney test. (**I**,**L**) WB analysis for Plin2 and Plin1 in *VL* biopsies of healthy subjects (**I**) and patients (**L**) of different age. Plin1 analysis indicates that samples are not contaminated with adipose tissue (AT). βtubulin is used as protein loading control. (**M**) WB analysis for PPAR-γ in *VL* biopsies of healthy subjects (He) and patients (Pa). (**N**) WB quantification of Plin2 expression performed using ImageJ software and normalized to βtubulin. Δ*p*<0.0001, one-way ANOVA test; ‡*p=0*.*0013*, Student’ *t*-test. (**O**) Representative WB analysis for Plin2 in male and female patients (Pa) of different age.

We first checked the presence of lipid droplets by using classic Oil Red O staining in 16 samples of muscle biopsy from patients, and we found an age-related increase in the positivity of the fibres to this staining (Figure 1A–C). To check whether the accumulation of lipid droplets is linked to Plin2 expression, we analysed the expression of Plin2 in the muscle of the same subjects used for Oil Red O staining. In IHC analysis, the whole section from each specimen was evaluated, with a mean of 18.75±9.79 fields/case (range 7.00-39.00). We observed that Plin2 increases with age (Figure 1D–F) and confirmed that Plin2 expression is specific of muscle fibres but not adipose tissue (AT), (Figure S1). We performed a semi-quantitative analysis of Plin2 positivity and assigned an arbitrary rank from 1+ to 3+. The subjects scoring 3+ (areas of strong Plin2 positivity at low magnification) resulted to be old patients, while those scoring 1+ (scattered positive droplets not visible at 10x magnification) were young patients (Figure 1G).

We further investigated Plin2 expression by real time RT-PCR and WB in muscle biopsies from healthy subjects (He) and patients (Pa). We were able to confirm that Plin2 expression is higher in old subjects, both healthy and patients (Figure 1H–L). Of note the levels of Plin2 protein were higher in patients than in healthy subjects, with a consistent accumulation in oldest subjects, suggesting that inactivity affects Plin2 expression (Figure 1I, L). The expression of PPARγ, the main positive transcriptional regulator of Plin2 [21] resulted consistent with Plin2 expression (Figure 1M). A densitometric quantification of WB bands allowed us to confirm that, among old subjects, patients have higher levels of Plin2 (Figure 1N, p= 0.005). The analysis was conducted considering the two sexes separately, and this allowed us to identify a significant difference between males and females in the group of young patients, where women express much higher levels of Plin2 compared to men (Figure 1N, O). This suggests that in young people a sex difference emerges only with physical inactivity, and that in old people the effect of sex disappears.

**Figure 2:**
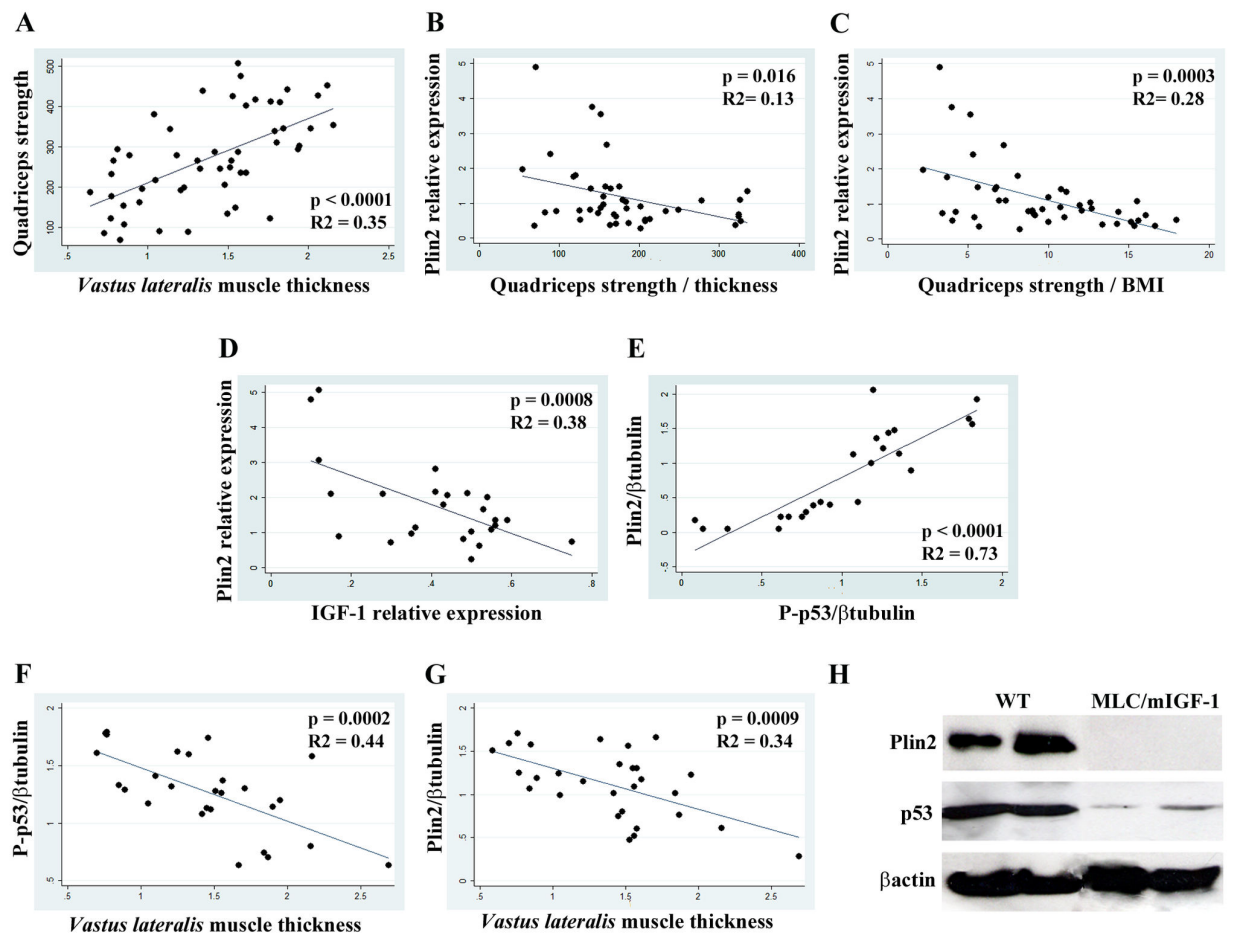
Plin2 association with muscle strength and thickness, and with IGF-1/p53 pathway. (**A**) Linear regression analysis in patients of quadriceps strength (expressed in Newton) and *VL* thickness. (**B**, **C**) Linear regression analysis in patients of Plin2 relative expression and quadriceps strength normalised for thickness or BMI, respectively. (**D**, **E**) Linear regression analysis in healthy subjects and patients of Plin2 and IGF-1 relative expression level (Real time RT-PCR), and of Plin2 and serine-20 phosphorylated p53 protein expression (relative band intensity), respectively. (**F**, **G**) Linear regression analysis in healthy subjects and patients of serine-20 phosphorylated p53 protein expression and *VL* thickness, and Plin2 and *VL* thickness, respectively. (**H**) Representative WB analysis for Plin2 and total p53 in wild type (WT) and transgenic IGF-1 (MLC/mIGF-1) old mice (23-25 months). βactin is used as protein loading control.

Ultrasound analysis allowed us to evaluate in patients *VL* muscle thickness, which resulted to be correlated with isometric quadriceps strength measured with dynamometer (Figure 2A). On the basis of this observation, we normalized the value of quadriceps strength by thickness and investigated the possible correlation with Plin2 expression. A significant inverse correlation was found (Figure 2B). This correlation was also present when muscle strength was normalised by BMI (Figure 2C). A similar trend was also found for healthy subjects (data not shown). Since IGF-1 is known for being a trophic factor for muscle also acting on lipolysis [22], and down-regulating PPAR-γ [23], we investigated whether Plin2 expression was correlated with IGF-1 expression in human *VL* samples. A significant inverse correlation was found (Figure 2D). As it is known that PPAR-γ can inhibit p53, an important mediator of muscle wasting [24], we also investigated the possible correlation of Plin2 expression and p53 activation, assessed as phosphorylation at serine 20. We found a positive correlation between the amount of phosphorylated p53 and Plin2 (Figure 2E). The level of phosphorylated p53 resulted also inversely correlated with *VL* thickness (Figure 2F), indicating that p53 activation is involved in muscle wasting. We then observed that also Plin2 protein levels had an inverse correlation with *VL* thickness (Figure 2G).

To further substantiate the hypothesis that the age-associated increase of Plin2 is involved in muscle weakness, we investigated the expression of Plin2 and p53 in both aged WT and transgenic mice of 23-25 months of age, overexpressing the local form of IGF-1 (mIGF-1) selectively in skeletal muscle [19]. These mice maintain the integrity of muscle mass and strength during aging [18]. Notably, Plin2 and p53 resulted much less expressed in the muscle of mIGF-1 mice than in WT littermates (Figure 2H), suggesting that mIGF-1 counteracts sarcopenia by modulating Plin2.

## Discussion

In this study we describe the different expression levels of Plin2, a IMTG associated protein, in the *VL* of young and old subjects, either healthy or with lower limb mobility impairment. In particular, we found that Plin2 is much highly expressed in old subjects, and mainly in patients where Plin2 is inversely correlated with *VL* thickness and quadriceps strength.

Plin2 has been found to be associated with the storage of triacylglycerols in skeletal muscle, blunting lipotoxicity-associated insulin resistance [13,14], but little is known about its function in the regulation of IMTG metabolism during aging. On the basis of these previous results, it could be expected that in old age, where insulin resistance takes place, the expression of Plin2 may be decreased. Surprisingly our data indicate that Plin2 expression is increased in old people and that this increase is particularly evident in patients with lower limb mobility impairment.

It is known that there are sex-dependent differences in Plin2 expression, in particular women are characterised by higher levels of Plin2. In our study such a sex difference is present only in young patients, but not in old patients nor in healthy subjects. This suggests that i) in young people a sex difference emerges only with physical inactivity, and ii) in old people the effect of sex disappears. This sex difference in young patients could be due to the effect of sex hormones that leads to the accumulation of adipose tissue in the hips and thighs of women.

Interestingly, our data suggest that Plin2 expression is an indicator of muscle weakness in patients with reduced mobility, either young or old. In these patients Plin2 expression is inversely correlated with *VL* muscle thickness and quadriceps strength, normalised by either *VL* thickness or BMI. These data suggest that Plin2 expression is associated with a decrease of muscle strength per unit of thickness, indicating that, as intracellular accumulation of fat in form of LDs increases, the muscle becomes intrinsically weaker (less strength per unit size).

**Figure 3:**
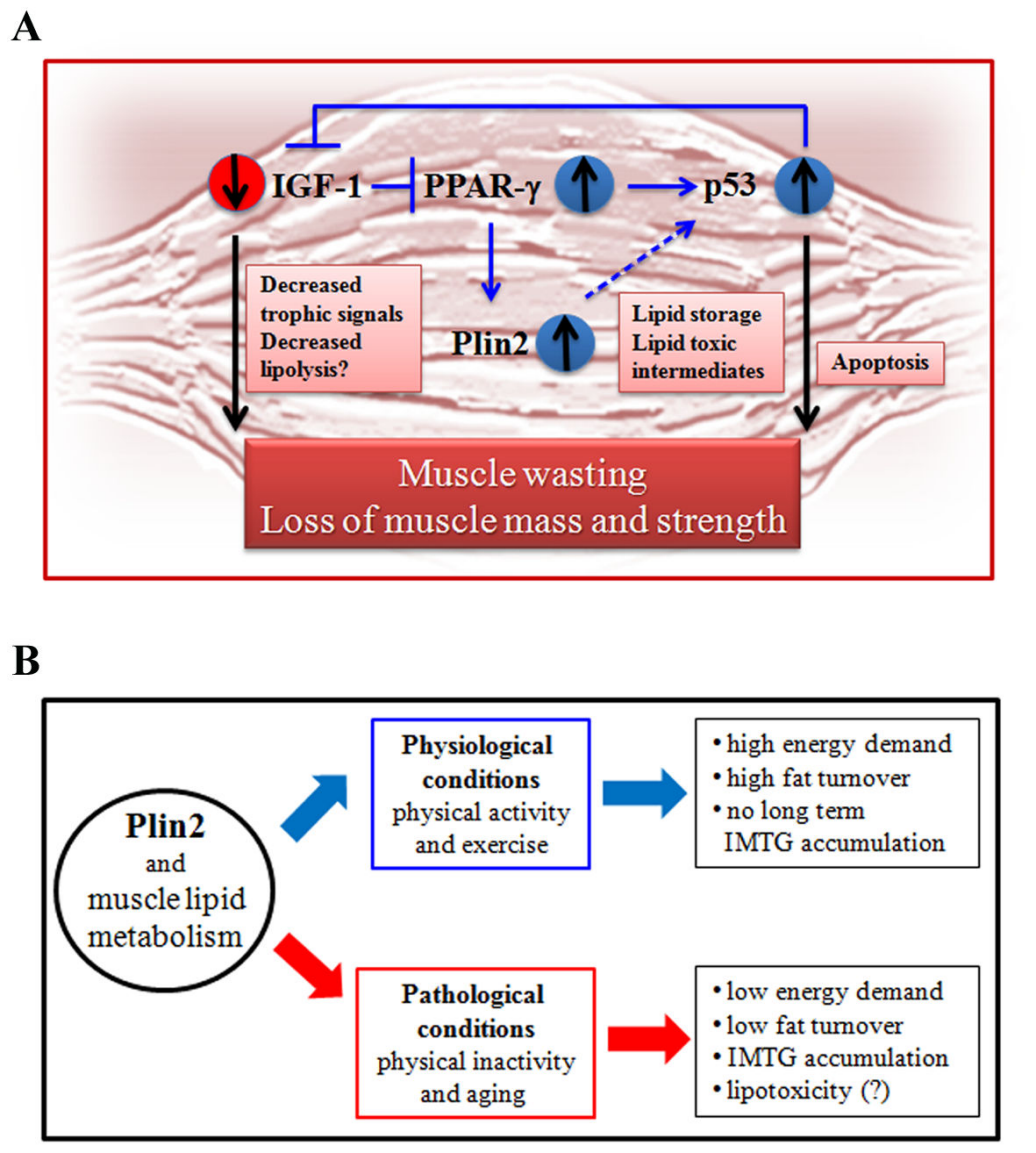
Schematic representations of the hypothetical role of Plin2 in muscle aging. (**A**) Proposed mechanism of action. Circled arrows indicate increased or decreased expression with aging. Plin2 could impact on p53 activation likely through indirect mechanisms (dashed arrow) possibly including the spill over of toxic lipid intermediates. (**B**) Hypothetical role of Plin2 in muscle physiology and pathology.

The effects of Plin2 on muscle metabolism are not totally understood. On one side, the expression of Plin2 appears to be beneficial as it has been associated with the utilization of IMTG during exercise [25]. On the other side, it is reported that pharmacological inhibition of fatty acids entry to mitochondria leads to an increased Plin2 expression [26]. Our data showing an increased level of Plin2 expression with aging and inactivity seem to be suggestive of an increased deposition of LDs rather than a mobilization of fatty acids subsequent to exercise. This consideration is in agreement with the role of LDs as buffer between fatty acid supply and use [16]. In our patients the physical inactivity due to a mobility limitation likely leads to a lower utilization of fatty acids in the muscle and then to an accumulation of LDs that we can detect as an increased expression of Plin2. Therefore it is conceivable that during aging, Plin2 expression is linked to fatty acid storage rather than utilisation, as it is known to occur in athletes [25]. It is known that fat accumulation in LDs is linked to an excessive flow of lipid intermediates leading to the up-regulation of PPARs and to an increased formation of oxidized lipid and ROS that cause mitochondrial dysfunction and activation of transcription factors [16], such as p53. In our samples we found indeed a striking correlation between the amount of phosphorylated p53 and Plin2. p53 is known to play a role in muscle wasting, due to its role in regulating apoptosis [27]. Moreover, p53 has been found to lead to skeletal muscle atrophy and muscle stem cell perturbation [28]. Proapoptotic p53 downstream genes such as puma, noxa, dr5 and bok have been also found to be more expressed in muscle of old animals with respect to young ones [29]. Therefore, it is likely that the accumulation of Plin2 is not detrimental *per se* but it should be rather interpreted as an attempt of the cells to get rid of excessive fatty acids, and we hypothesise that p53 activation in skeletal muscle, possibly triggered by these toxic lipid intermediates can have a role in the decrease of muscle thickness observed in old patients. This hypothesis is summarised in Figure 3A, even if other possible mechanisms cannot be excluded. As an example, we observed that PPAR-γ, the main transcription factor for Plin2 is more expressed in samples with high Plin2 levels, and it is reported that PPAR-γ can activate p53, at least in some experimental models [24,30,31].

It is known that IGF-1 has lipolytic effects on muscle tissues [22]. In our samples an inverse correlation between the expression of IGF-1 and Plin2 at the level of muscle was found. We can therefore speculate that when IGF-1 decreases, as in old age, fatty acid degradation also decreases, and Plin2 expression increases. Moreover, IGF-1 and p53 are part of a feedback loop in which, when p53 is activated, the effects of IGF-1 are blunted because of a p53-mediated suppression of IGF-1R transcription, an increase in the expression of IGF1-BP3, PTEN and TSC2 which antagonise the signalling of IGF-1 to Akt and mTOR [32]. The data we obtained on mIGF-1 transgenic mice are in agreement with these previous findings and support the hypothesis that IGF-1 and p53 are crucial players of muscle sarcopenia, as these animals that were previously reported to maintain the integrity of muscle mass and strength during aging without accumulation of body fat [18], resulted to have little or no expression of both Plin2 and p53.

To conclude, we hypothesise that an increased Plin2 expression in the muscle has different effects according to the age and the level of activity of the subjects: while in physiological conditions (young healthy subjects) Plin2 is associated with lipid turnover and consumption, during aging or inactivity, its expression is associated with a pathological condition that leads to IMTG accumulation and lipotoxicity with p53 activation and decreased muscle mass and strength, a condition that can be counteracted by IGF-1 (Figure 3B). The precise molecular mechanisms underlying this phenomenon are still not clear and deserve further investigations.

## Supporting Information

Table S1 **General characteristics of the study population: healthy subjects.** *N* = number of participants. Values are means ± SD.
(DOCX)

Table S2 **General characteristics of the study population: patients with limited lower limb mobility.** *N* = number of participants. Values are means ± SD.
(DOCX)

Figure S1 **Tissue-specific localization of Plin1 and Plin2 expression in VL.** (**A**, **B**) Immunohistochemical analysis of three µm-thick sections from *VL* muscle showing the staining pattern (brown colour) of Plin2 (**A**) and Plin1 (**B**). Note that Plin1 is localized in the adipocyte membranes while Plin2 is localized within the myofibres. Magnification 20x. (**C**) Real time RT-PCR expression level of Plin1 and Plin2 in skeletal muscle from 11 patients. The relative expression levels of Plin1 and Plin2 genes in skeletal muscle were normalized comparing their expression in the adipose tissue of the same patients used as calibrator. *p<0.0001. (**D**) Representative western blots of Plin1 and Plin2 in skeletal muscle (M) and adipose tissue (AT) of 5 patients (P1-P5).
(TIF)
