# Supplementary material for: Increased Plin2 Expression in Human Skeletal Muscle Is Associated with Sarcopenia and Muscle Weakness
Source: PLoS One. 2013 Aug 15;8(8):e73709. doi: 10.1371/journal.pone.0073709 (PMC3744478; doi:10.1371/journal.pone.0073709)
Supplement: Table S1 — General characteristics of the study population: healthy subjects. N = number of participants. Values are means ± SD. (DOCX) [file pone.0073709.s001.docx]

**Table S1**

| **HEALTHY GROUP** | | |
| --- | --- | --- |
| **Characteristics of subjects** | **< 40 yrs**  *N* = 15 | **> 70 yrs**  *N* = 30 |
| Gender | 8 F – 7M | 16 F – 14M |
| Age (yrs) | 21.6 ± 2.23 | 73.37 ± 2.66 |
| Weight (Kg) | 72.87 ± 13.34 | 73.8 ± 11.44 |
| Height (cm) | 176 ± 8.13 | 172 ± 6.87 |
| BMI | 23.51± 3.69 | 25.01 ± 3.21 |
