# Supplementary material for: Increased Plin2 Expression in Human Skeletal Muscle Is Associated with Sarcopenia and Muscle Weakness
Source: PLoS One. 2013 Aug 15;8(8):e73709. doi: 10.1371/journal.pone.0073709 (PMC3744478; doi:10.1371/journal.pone.0073709)
Supplement: Table S2 — General characteristics of the study population: patients with limited lower limb mobility. N = number of participants. Values are means ± SD. (DOCX) [file pone.0073709.s002.docx]

**Table S2**

| **PATIENTS’ GROUP** | | |
| --- | --- | --- |
| **Characteristics of subjects** | **< 40 yrs**  *N* = 20 | **> 70 yrs**  *N* = 20 |
| Gender | 7 F – 13M | 11F – 9M |
| Age (yrs) | 33 ± 4.79 | 82.1 ± 7.89 |
| Weight (Kg) | 76.6 ± 15.65 | 66.25 ± 15.97 |
| Height (cm) | 169 ± 11.77 | 159 ± 11.30 |
| BMI | 26.52 ± 3.74 | 25.71 ± 4.32 |
