# Supplementary figures and images for: Increased Plin2 Expression in Human Skeletal Muscle Is Associated with Sarcopenia and Muscle Weakness
Source: PLoS One. 2013 Aug 15;8(8):e73709. doi: 10.1371/journal.pone.0073709 (PMC3744478; doi:10.1371/journal.pone.0073709)

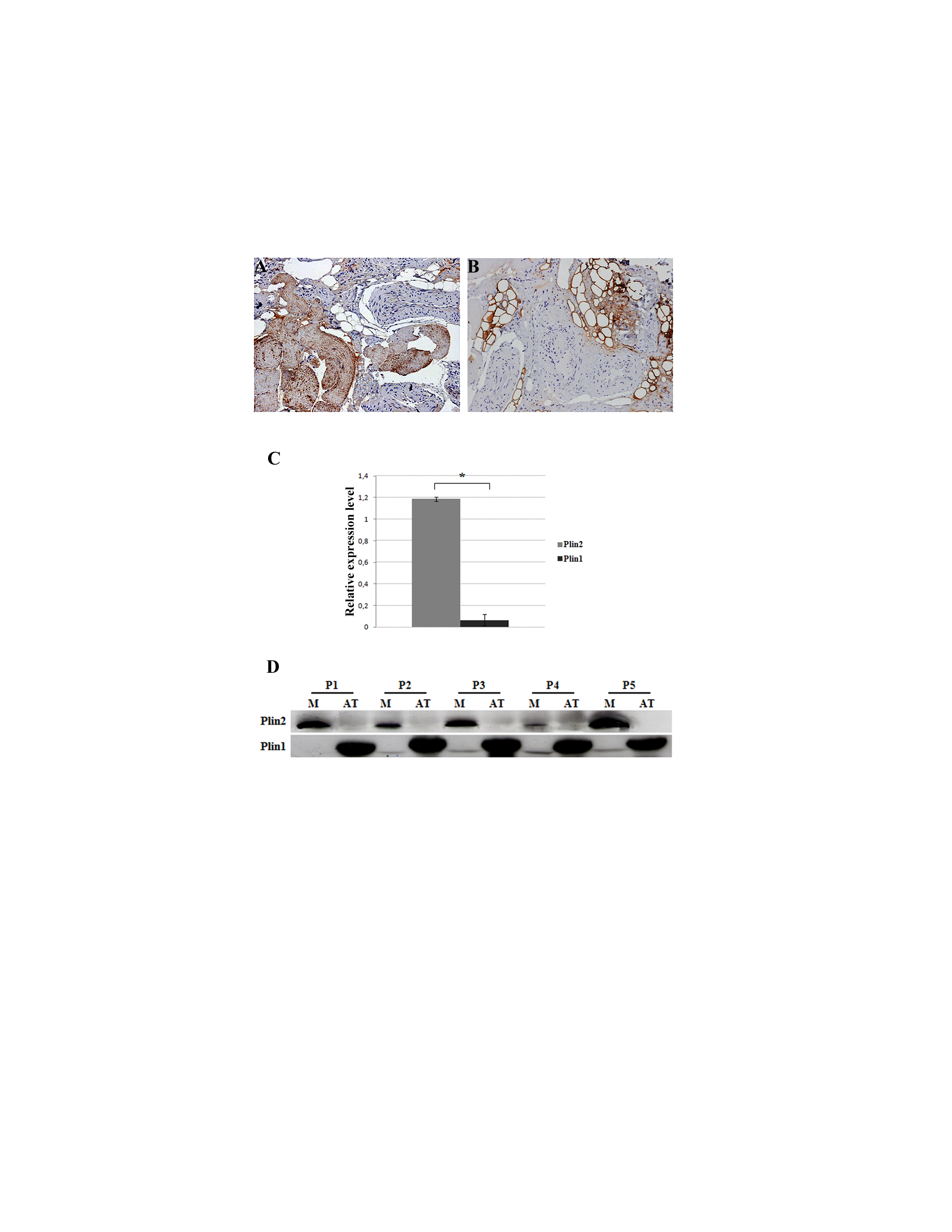

Supplement: Figure S1 — Tissue-specific localization of Plin1 and Plin2 expression in VL. (A, B) Immunohistochemical analysis of three µm-thick sections from VL muscle showing the staining pattern (brown colour) of Plin2 (A) and Plin1 (B). Note that Plin1 is localized in the adipocyte membranes while Plin2 is localized within the myofibres. Magnification 20x. (C) Real time RT-PCR expression level of Plin1 and Plin2 in skeletal muscle from 11 patients. The relative expression levels of Plin1 and Plin2 genes in skeletal muscle were normalized comparing their expression in the adipose tissue of the same patients used as calibrator. *p<0.0001. (D) Representative western blots of Plin1 and Plin2 in skeletal muscle (M) and adipose tissue (AT) of 5 patients (P1-P5). (TIF) [file pone.0073709.s003.tif]
